# Explaining the uptake of paediatric guidelines in a Kenyan tertiary hospital – mixed methods research

**DOI:** 10.1186/1472-6963-14-119

**Published:** 2014-03-10

**Authors:** Grace W Irimu, Alexandra Greene, David Gathara, Harrison Kihara, Christopher Maina, Dorothy Mbori-Ngacha, Dejan Zurovac, Migiro Santau, Jim Todd, Mike English

**Affiliations:** 1 Department of Paediatrics and Child Health, College of Health Sciences, University of Nairobi, P.O. Box 19676–00202, Nairobi, Kenya; 2 Centre for Geographic Medicine Research – Coast, KEMRI/Wellcome Trust Research Programme, P.O. Box 230 Kilifi, Nairobi, Kenya; 3 P.O. Box 43640–00100, Nairobi, Kenya; 4 Child Health, University of Dundee, Dundee, UK; 5 Kenyatta National Hospital, P.O. Box 20723–00202, Nairobi, Kenya; 6 Nuffield Department of Clinical Medicine, Centre for Tropical Medicine, University of Oxford, CCVTM, Oxford, OX3 7LJ, UK; 7 Center for Global Health and Development, Boston University, Boston, MA 02118, US; 8 Division of Child Health, Ministry of Health, Nairobi, Kenya; 9 London School of Hygiene and Tropical Medicine, Keppel Street, WC1E 7HT, London, UK; 10 Department of Paediatrics, University of Oxford, Oxford, UK

**Keywords:** ETAT+, Ethnographic, Guidelines, Implementation, Performance, Mixed methods research, Hospital leadership, Complex adaptive system

## Abstract

**Background:**

Evidence-based standards for management of the seriously sick child have existed for decades, yet their translation in clinical practice is a challenge. The context and organization of institutions are known determinants of successful translation, however, research using adequate methodologies to explain the dynamic nature of these determinants in the quality-of-care improvement process is rarely performed.

**Methods:**

We conducted mixed methods research in a tertiary hospital in a low-income country to explore the uptake of locally adapted paediatric guidelines. The quantitative component was an uncontrolled before and after intervention study that included an exploration of the intervention dose-effect relationship. The qualitative component was an ethnographic research based on the theoretical perspective of participatory action research. Interpretive integration was employed to derive meta-inferences that provided a more complete picture of the overall study results that reflect the complexity and the multifaceted ontology of the phenomenon studied.

**Results:**

The improvement in health workers’ performance in relation to the intensity of the intervention was not linear and was characterized by improved and occasionally declining performance. Possible root causes of this performance variability included challenges in keeping knowledge and clinical skills updated, inadequate commitment of the staff to continued improvement, limited exposure to positive professional role models, poor teamwork, failure to maintain professional integrity and mal-adaptation to institutional pressures.

**Conclusion:**

Implementation of best-practices is a complex process that is largely unpredictable, attributed to the complexity of contextual factors operating predominantly at professional and organizational levels. There is no simple solution to implementation of best-practices. Tackling root causes of inadequate knowledge translation in this tertiary care setting will require long-term planning, with emphasis on promotion of professional ethics and values and establishing an organizational framework that enhances positive aspects of professionalism. This study has significant implications for the quality of training in medical institutions and the development of hospital leadership.

## Background

Effective and cost-effective best-practices in management of the seriously sick child have existed for decades, yet knowledge translation, in the form of practice uptake and its maintenance, is challenging [1-4]. As part of efforts to promote uptake of good practices in district hospitals in Kenya the Ministry of Health, in collaboration with stakeholders, developed evidence-based clinical practice guidelines (CPGs) – named ‘Basic Paediatric Protocols’ [5]. The guidelines aimed to improve paediatric emergency and admission care in the initial 48 hours of hospitalization. A 5-day training programme for dissemination of the CPGs, dubbed ‘Emergency, Triage, Assessment, Treatment *Plus* admission care (ETAT+)’ was also developed [5,6]. The CPGs and ETAT + were popular and demand grew even in higher-level hospitals including Kenyatta National Hospital (KNH) which is the largest referral and teaching hospital in Kenya [7].

We have elsewhere reported quantitative and qualitative research that were carried out concurrently to determine and explore uptake of CPGs and ETAT + in KNH. In the quantitative study we described a before and after study examining changes in health workers’ performance following the dissemination of the CPGs in KNH [8]. In the qualitative work, using an ethnographic approach and theoretical perspective of participatory action research, we provided a context-driven description of the implementation process including the facilitators of and barriers to this process [9]. Each report contributes to our knowledge of the implementation process, by providing some understanding of the extent of change and how this varied. It also demonstrated how dynamic institutional effects related to the context and organization of the hospital influenced the success. A limitation of these prior analyses is that they identify immediate operational constraints (micro-level factors) rather than getting to the root cause of the factors that influenced adoption of best-practices.

As our interest is to try and fully understand what might be effective implementation strategies we also planned, *a priori*, to use these data in a mixed methods research analysis that recognizes the complexity of the intervention, the barriers and contexts and their interplay. To achieve this, we conducted an interpretive (analytic) integration of the quantitative and qualitative data to derive meta-inferences that provided an overarching explanation of the study results that reflect the complexity and the multifaceted ontology of the phenomenon studied [10].

In this paper, we report trends in change of different aspects of the health workers’ performance examined over a 5 year period spanning a pre-intervention phase to the end of a period of intervention reinforcement. We synthesise the results of the two research paradigms into a single discussion section and demonstrate how the paradigms worked synergistically to provide a whole that is greater than the sum of its parts and increase the knowledge yield [10,11]. Finally we discuss the implications of the results of the overall study.

## Methods

### Study design

**Figure 1:**
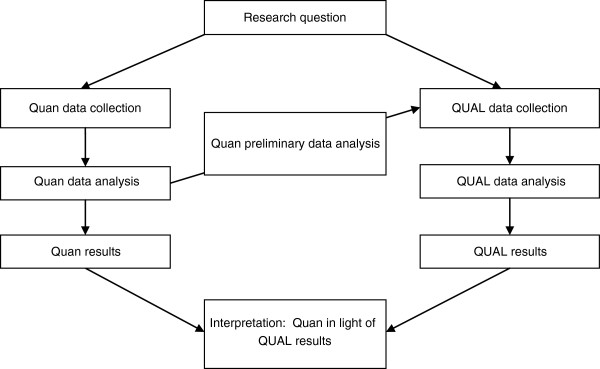
Visual presentation of research procedures demonstrating the levels of integration.

This was a hospital–based, pragmatic study that utilized mixed methods research based on the theoretical perspective of participatory action research (PAR). The quantitative component was an uncontrolled before and after design that included exploration of the intervention dose-effect relationship [8]. The qualitative component adopted a PAR approach based on traditional ethnographic research methodology [9]. Integration of the two research paradigms occurred at the level of formulation of the research question, at the methodology level where the preliminary results of the quantitative research informed the PAR activities and finally at the level of interpretation of the results. Priority was given to the qualitative (QUAL) over the quantitative (Quan) research (Figure 1).

### Study site

This research was conducted in Kenyatta National Hospital (KNH), the largest tertiary hospital in Kenya and the teaching hospital for the University of Nairobi Medical School. We have previously described KNH [8,9]. In brief, KNH’s vision is ‘*To be a regional centre of excellence in the provision of innovative and specialized health care*’. KNH’s mandate is to provide specialized health care, to provide facilities for research and training of health professionals, and to participate in national health planning and policy. KNH is the second largest hospital in Africa with a bed capacity of 1800; it has four general paediatric wards each with a bed capacity of 60, though bed occupancy is often over 100%. There are 14,000 paediatric admissions annually. Majority (65%) of patients admitted with acute childhood illnesses are referred from primary care facilities, 20% are direct self-referrals and only 15% are referred from public or private hospitals (unpublished data).

The clinical service delivery unit of general paediatrics consisted of 25 consultants of whom 15 were academics and 10 employees of KNH. In this paper we refer to all the paediatricians as consultants being the name typically used in this hospital. In line with the hospital’s vision, 22 of the paediatricians were professors and/or subspecialists such as paediatric nephrologists and cardiologists. Each ward had 5–8 paediatricians, 5–8 paediatric trainees and a nutritionist. There were approximately three to four nurses on duty each working shift.

### Procedures

We have presented the methods for both qualitative [9] and the quantitative [8] research components separately to allow detailed description. The quantitative research examined hypotheses with a focus on assessing the relation between interventions and uptake of best-practice recommendations. The qualitative research was explanatory with a strong emphasis on ‘thick description’ of the phenomena [12], a thematic focus on the implementation process of best-practice recommendations and why additional reinforcement interventions worked or did not work. The qualitative data were largely in the form of 18 months’ diary entries of participant observations and reflective notes. The data collection from the two research methods was concurrent.

### Approach to data analysis

We present data analysis in two parts; first our exploration of the trends for changes in different aspects of the health workers’ performance and then our approach to interpretive integration of the quantitative and qualitative data sets.

### Trends for change in different aspects of health workers’ performance

The intervention was delivered and evolved over 18 months. The contextual factors and effect modifiers arising as a result of the learning during the PAR must then be appreciated as parts of the intervention. Interpretation of the ‘effect size’ from our before and after intervention study should therefore be more nuanced. We therefore explored the trends for changes (2005–2009) in greater depth by graphically displaying the six monthly performances of 13 quality indicators with bars indicative of the 95% confidence intervals (CI) around an estimate of the mean performance. We have previously described the development and definition of the indicators [8,9]. In brief, the definitions of the indicators were based on KNH’s adaptation of WHO/Kenya case management guidelines [5,13]. An indicator was considered achieved or correct if the care was consistent with CPGs, ETAT + recommendations or staff consensus (Table 1). We excluded two indicators that evaluated follow-up care of the patients who died in the first 48 hours after admission because the small number of these patients precluded meaningful analysis.

**Table 1:** Definition of the composite indicators of processes of care for each disease (doi:10.1371/journal.pone.0039964.t001.)

**Domain of care**	**Criteria for considering the composite indicator achieved**	**Pneumonia**	**Dehydration**	**Severe malnutrition**
**Assessment**	Patient adequately assessed if all the following signs are assessed	Level of consciousness ability to drink^a^, cyanosis, lower chest wall indrawing and respiratory rate	Level of consciousness, pulse character^b^, ability to drink^a^, sunken eyes and skin turgor (and duration of skin fold to return)	Oedema, and weight for height Z-score or visual assessment of degree of severe wasting
**Classification**	Consistent with CPGs/ETAT + if any the corresponding terms are used	Very severe pneumonia, severe pneumonia,	Shock, severe dehydration, some dehydration and no dehydration	Severe malnutrition, oedematous malnutrition, protein energy malnutrition, marasmic kwashiorkor, kwashiorkor marasmus
**Treatment**	Consistent with CPGs if the following key treatment was prescribed at the correct dose and frequency (and duration for rehydration therapy)	Crystalline penicillin 50,000 units/kg/dose × 4 per day (+/-20%) and/or Gentamicin 7.5 mg/kg/day × 1 per day (+/-20%)	Hartman’s solution^c^ at 80–120 mls per kg if not given bolus for shock management or 56–120 mls per kg if given bolus for shock management given over 5–6 hours for patients ages 2–11 months and 2.5-3 hours in patients aged 12–59 months	100-130 mls/kg/day (+/-20%) of F75^d^
**Follow**-**up care**	Consistent with WHO/Kenya guidelines as adapted by the hospital staff	Evidence that doses of Crystalline penicillin were given as prescribed in the first 48 hrs of admission^e^	Evidence that intravenous fluid (IV) therapy for severe dehydration was monitored	Evidence that intake of feeds for severe malnutrition was monitored^e^

^a^Patients documented to have altered consciousness were assumed that they are not able to drink if ability to drink is not documented while patients documented in the history as able to drink were assumed to have the sign ‘able to drink’.

^b^Patients documented as able to drink or alert were assumed not to have a weak pulse if pulse character was not documented.

^c^If dextrose added, correct if given at 2.4-6.0 mg/kg/min (approximates dextrose requirement for a sick child 3-5 mg/kg/min; +/-20%).

^d^Was either a manufactured product (depending on the availability) or milk-based solution prepared in the hospital that provided 75 kcal and 0.9 g of protein/100 ml.

^e^Initial treatment is considered given on time if it is given within 12 hours of admission on the ward.

In an attempt to relate the performance of quality of care indicators to intensity and coverage of ETAT + training, we divided the entire study into four periods: i) Period 0 - pre-intervention period; January to December 2005, ii) Period 1 - piloting of ETAT + training materials; January to December 2006, iii) Period 2 - formal ETAT + training; January 2007 to June 2008, and iv) Period 3 - period of PAR; July 2008 to December 2009. Change in performance was considered of potential interest if the 95% CI of the six monthly mean performance measures suggest statistically significant changes between measurements.

### Interpretive integration

The first step was to make a summary of the main findings from the quantitative and qualitative data sets. Then, we examined the fit of hypotheses generated from qualitative research across composite quality indicators derived from four domains of care (assessment, classification, treatment and treatment administration) depicted in the quantitative data [8]. We took notes and reflected on the variations of performance across the target diseases. We moved back and forth constantly between the two data sets asking ourselves questions to interrogate the data by using a framework adapted from Strivastava and Hopwood’s work [14] (Table 2). We acknowledged that the structure can define the scope of an agent’s ability to act and agency may also have the ability to change the structure [15].

**Table 2:** **Frame work for interrogating the data sets - adapted from Strivastava and Hopwood (2009)**[14]

**Theme**	**Approach to interrogation of the data sets**
Relevance of the data to the research question	What are the data telling us in reference to the research questions?
	What is it we want to know according to the research questions and theoretical points of interest?
Variation of data	How does the performance vary across and within domains of care?
	What aspects of the qualitative data can explain this variation and are there other factors contributing to this relationship?
	What does this imply in regard to achieving the quality indicators in a certain domain?
	What is the dialectal relationship between what the data are telling us and what we want to know?
Relevance of the data to the context	How will it be understood by the health professionals and the hospital management?
	What do we want to know about the interconnectedness of the institution and individual professionals?

The integration was an iterative process; requiring frequent revisiting of the databases and gathering more information as additional questions emerged and new connections and deeper understanding of the data occurred. The findings were related to other empirical studies in this field, theoretical frameworks, our experiences and interdisciplinary backgrounds [16]. Social cognitive theory [17,18] and complex adaptive system theory [19,20] provided a framework for understanding our data and root cause analysis.

### Ethics statement

Ethical approval was provided by the Kenyatta National Hospital/University of Nairobi Ethics and Research Committee (reference number KNH-ERC/01/480).

## Results

We have described in detail the performance of 15 key quality indicators in the pre-intervention period and post-intervention periods elsewhere [8]. The trend for change for exemplar indicators over 5 years (2005–2009) in relation to the stage of implementation is described below. We have described the results of the qualitative component of this research elsewhere [9]. In brief, resource mobilization, relevance of ETAT + to routine work and emergence of a champion of change facilitated implementation of best-practices. Barriers to implementation of best-practices are summarized in Table 3.

**Table 3:** **Barriers to implementation of best**-**practices observed in the participatory action research**

**Theme**	**Implications**
Mismatch between hospital’s vision and reality	The hospital strategic planning was based on its vision to provide innovative and specialized health care contrary to the reality that majority of patients had common acute illnesses that did not require specialized care. There was a mismatch of infrastructure and the skill mix of the workforce did not sufficiently match the patient’s needs.
Poor communication	Poor communication was compounded by a centralized administrative system and limited forums where working relationships could be discussed thus hampering knowledge sharing.
Limited objective measures for evaluating quality of clinical care	Absence of more objectively assessed measures of patients’ care meant inadequacies in self- regulation could arise and persist without notice.
Limited capacity for strategic planning.	Inadequate structures to optimize efficiency of service delivery.
Inadequate management skills to introduce and manage change.	Unwillingness to do things differently reflected a general negativism towards innovation and limited ability of the managers to articulate, supervise and guide change efforts.
Hierarchical relationships among the staff and patients	Passage of knowledge was largely unidirectional with lower cadres being the recipients. Doctors as well other health workers maintained their primacy in care of patients and protected their profession.
Inadequate adaptation of ETAT + to the local context.	Among all cadres, there was inadequate knowledge in some basic procedures that were not the focus of ETAT+. Some of the existing job aids were outdated and did not permit staff to adopt best-practices.

### Trends for change in different aspects of health workers’ performance

For the indicators that showed improved performance over time, the improvement was not linear and was characterized by improved and occasionally even declining performance. Furthermore, the response of the indicators to intervention was different; with some of the indicators showing a rapid response while others showed a delayed response. Below is a description of the major events that took place as the intervention evolved depicting the quality indicators that showed a significant change during the corresponding study periods.

#### Period 0 (Pre-intervention period)

**Figure 2:**
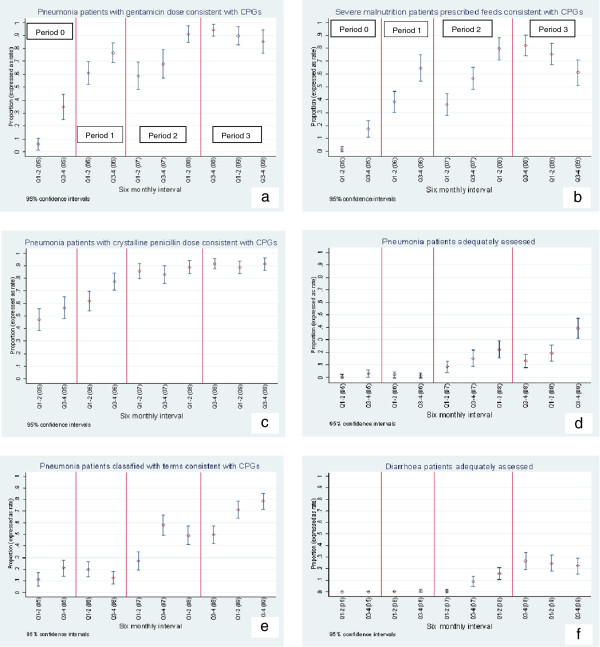
**Trend for change for proportion of patients who achieved key quality indicators across periods of intervention. *a***-***f***: Trend for change for proportion of patients who achieved key quality indicators across periods of intervention Period 0 – Pre-intervention period Period 1- Piloting ETAT + training materials Period 2 – Formal scaling up of ETAT + Period 3 – Period of PAR.

Performance of indicators for prescription of gentamicin for pneumonia and prescription of feeds for severely malnourished patients demonstrated a significant improvement in period 0. This change coincided with the Child Health Evidence Week in June 2005, a forum attended by some KNH consultants and trainee paediatricians in which the importance of prescribing correct drug dosages was highlighted [6]. Both indicators showed further improvement during the formal scale-up of ETAT + training. The PAR activities did not have a substantive effect on the gentamicin indicator. Moreover, some deterioration was observed in the performance of feed prescriptions (Figure 2a,b).

#### Period 1 (Piloting ETAT + training materials)

Prescription of crystalline penicillin for pneumonia patients improved rapidly in period 1 suggesting a link with piloting of the ETAT + training and small-scale distribution of CPGs booklets (to trainee paediatricians) and influence of early adopters. This performance was maintained during the rest of the study period and no further improvement was noted as a result of formal ETAT + training and PAR (Figure 2c).

#### Period 2 (Formal scale-up of ETAT+)

**Figure 3:**
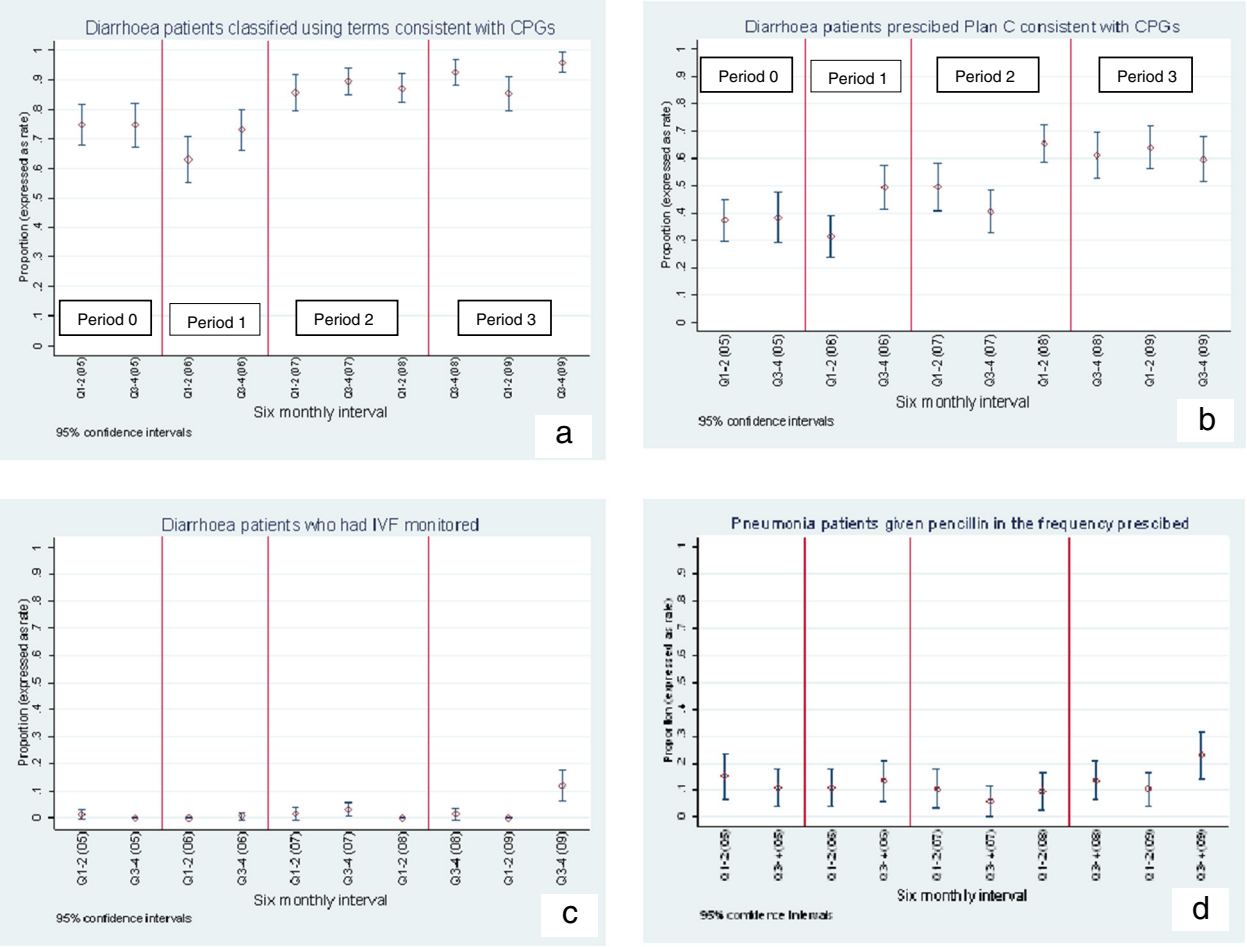
**Trend for change for proportion of patients who achieved key quality indicators across periods of intervention. *a***-***d***: Trend for change for proportion of patients who achieved key quality indicators across periods of intervention Period 0 – Pre-intervention period Period 1- Piloting ETAT + training materials Period 2 – Formal scaling up of ETAT + Period 3 – Period of PAR.

Five key indicators showed initial response that may be attributed to the gradual rolling out of the formal training. These included two indicators for pneumonia management (assessment and classification) and all the three indicators for diarrhoea management (assessment, classification and fluid prescription) (Figures 2d-f and 3a,b).

#### Period 3 (Period of PAR)

Major activities during PAR included audit and feedback, tailored educational sessions and reorganization of service delivery [9]. At the commencement of the PAR four indicators had not shown any improvement in performance. Despite attempts to promote uptake of the best-practices during the PAR only one of these indicators, monitoring of administration of intravenous fluid therapy improved, though the performance was still poor at the end of the study period (Figure 3c). Surprisingly, only two indicators (assessment and classification of pneumonia) demonstrated an augmented response during the period of PAR (Figure 2d,e). Except for prescription of feeds that showed deterioration in performance, other indicators that had previously improved maintained their performance.

Despite ETAT + training and promotion of uptake of best-practices, four indicators failed to show any significant improvement during the entire five year period (2005–2009). These included assessment and classification of severe malnutrition, administration of crystalline penicillin and monitoring of intake of feeds for the malnourished children. Among them only classification of severe malnutrition had good performance in the pre-intervention period. We display graphically the trend for change for the indicator of administration of penicillin as an example of indicators that showed no significant change (Figure 3d).

## Findings of the interpretive integration and discussion

Our interpretive integration of the quantitative and the qualitative data yielded six major themes that explained the uptake of CPGs and ETAT + recommendations in KNH. These themes were: i) knowledge and clinical skills, ii) commitment to continued improvement, iii) professional role models, iv) professional integrity, v) work in teams, and vi) mal-adaptation to institutional pressures.

### Knowledge and clinical skills

Professionals in this setting faced challenges in keeping knowledge and skills updated. However, the challenge of accessing knowledge in a low-income country like Kenya is different from that described in a high-income country where there is so much information and knowledge available that professionals ‘risk drowning in it’ [21]. Within KNH and UoN accessibility of knowledge was a problem. In addition, professionals had a culture that did not sufficiently support self-reading directed at improving practice and the training curriculum rarely encouraged trainee paediatricians to seek new information on patients’ care [22]. Accordingly in Kenya, as reported previously, problems in professionals’ behaviour are linked to problems in medical knowledge [4].

Clinicians are believed to be sensitive to threats of loss of clinical autonomy by using guidelines [23]. However we observed that clinicians in KNH appreciated the guidelines, a finding consistent with Sheaff *et al*’s work in the UK [24]. This paradoxical support for guidelines but failure often to promote their application we suggest was due to the fact that such guidelines and ETAT + provided a shortcut to being knowledgeable and an ability to teach evidence-based medicine with minimal effort. As a result, an important professional value was perceived as achieved – to be supportive of evidence-based guidelines was to be doing the right thing - this contributed to the acceptance of the CPGs in principle but not necessarily in practice.

The management of serious common illnesses, the focus of the CPGs, was however perceived as a simple task and was often therefore not given due attention by the senior professionals despite the major contribution of these diseases to mortality in KNH. Indeed there was a tendency to equate valued knowledge with the rarity of a medical condition. In fact, awareness of the ‘fine print’ medical diagnosis and management was perceived as more important than knowledge applicable to the burden of patients’ needs. The attitude expressed was that knowledge of ‘simple’ diseases does not define a paediatrician; this should have been mastered in basic training. However, with such illnesses ‘forgotten’ there was no one to teach such mastery; as those expected to teach also had limited knowledge on local and international recommendations for management of the common illnesses.

Mechanisms that might address such problems, such as appropriate knowledge management linked with professionals’ interactions, were not considered a priority in KNH. There were rigid inter-professional boundaries and a low value attached to socialization processes that hindered knowledge transfer. Although the approach to intervention aimed to address these hurdles through hospital audit and multi-professional feedback, considerable barriers to this resulted in little success in breaking down ‘silos’ [9].

### Commitment to continued improvement

Inadequate normative commitment of the long senior serving staff, who provided interpersonal support and role modelling, also enhanced the resilience of the pre-existing KNH norms and values that undermined change efforts. Professionals did not provide sufficient order-generating rules (standards) to help operationalize the best-practice recommendations and promote change. Such rules and standards, when codified, would include policies on appropriate documentation practices, for example for follow-up care of the seriously sick child. Such formal and informal rules and standards were however largely absent and it appeared that the introduction of CPGs did not promote real accountability of the professionals for the quality of care delivered. This was despite the fact that quality targets were collectively set in a consensus approach [9].

Thus, there were minimal active efforts among the senior staff to influence the uptake of best-practices, despite them having the necessary autonomy to exercise legitimate control over the conduct of their work and change working conditions as demonstrated by a champion of change in the PAR [9].

### Professional role models

Consultants’ everyday behaviour, including demonstrating expertise and the practice of ethics and commitment, is the living demonstration of professionalism. Patients’ reviews by consultants provide junior staff opportunities to observe diverse real-life narratives and engage in small-group discussions that can encompass and enact many conventional aspects of such professionalism. This experiential learning provides opportunities for thoughtful reflection and clarification of professional practice roles amongst trainees and has the potential to bridge the theory and practice gap [25]. The benefits of these interactions were, however, not exploited optimally in KNH because competing priorities limited consultants’ time and efforts to participate in such ‘informal’ curricular activities.

In contrast, exposure to negative role models increases the likelihood of trainees becoming cynical and adopting negative professional values. As a consequence, inadequate consultants’ commitment to ward-rounds and to improve care adversely influenced experiential learning. In general, therefore, adopted mechanisms to introduce ‘change’ were limited to formal, didactic sessions or meetings with little consideration given to the differences and applicability of different mechanisms of sharing both knowledge and values [26,27].

### Professional integrity

It appeared that many positive facets of professionalism were abandoned while the consultants were within the role context of the tertiary hospital. This contrasted with more positive expressions of professionalism in contexts outside the institution - within private practices or where consultants were engaged in research or leadership of national health programmes. In these contexts, the consultants appeared to be loyal to professional goals and observant of professional values, while the structure of KNH reduced the agents’ desire and ability to act professionally. A similar double standard was seen amongst other staff including trainee paediatricians and nurses. Such contrasts suggest that the contextual influence of KNH is very powerful and indicates that interventions targeting behaviour of individuals are likely to have limited success in the absence of major organizational change.

### Work in teams

In the KNH context, poor teamwork could be attributed to the lack of role clarity, hierarchical relationships, a centralized and cadre-specific administrative system, poor communication, and insufficient fora where working relationships could be discussed. All these determinants inhibited knowledge sharing across professional boundaries. Inadequate engagement of the senior staff in decision-making made them identify less with the hospital services. In addition, such personnel acted in a system that did not have a mechanism to hold the staff accountable for their actions or inactions while paternalistic relationships with the patients precluded this group holding them to account.

#### Teamwork within disciplines

Performance in administration of treatment and conduct of patients’ regular clinical and nursing reviews are illustrative of problems of collaboration among professionals of the same discipline. These activities represent an aggregate of tasks performed by several interdependent care providers in the first 48 hours of admission. For effective review of patients’ progress after admission on the ward, knowledge of the clinical state of the patient during previous reviews is required. Poor documentation of these activities suggests that clinicians did not recognise the interconnectedness of these tasks, or if recognised, they did not consider it important to provide co-workers with information needed to allow meaningful assessment of patients’ progress. Thus, on admission the signs documented were those perceived to be just adequate to allow one to make a diagnosis, fulfilling the needs of the primary clinician, but inadequate to allow the patient’s progress to be adequately assessed by subsequent clinicians. This was likely sustained by the fact that fora to discuss work processes were rare.

Multi-disciplinary care was inadequately developed in KNH. If sub-optimal care was observed it was the norm of neither the nurses nor doctors to question. For example, there was limited documentary evidence that crystalline penicillin for treatment of pneumonia, intravenous fluid for severe dehydration, or feeds for the malnourished children were given to the patients as prescribed. This problem, while widely known to all professionals and highlighted during audit feedback meetings continued to be seen by clinicians as a ‘nursing problem’ and not of their concern. Inadequate role clarification exacerbated the situation. For example, it was not clear whether it was the nurses’, nutritionists’ or clinicians’ responsibility to take height and calculate the Z-score (for diagnosis of malnutrition) or oversee monitoring of feed intake. No group ultimately accepted responsibility resulting in insignificant change in this practice. In fact, perceiving problems as the responsibility of ‘other professions’ absolved one from personal responsibility and undermined staff’s self-efficacy to improve care even in their own ‘territory’.

#### Paternalistic relationships with the patients

It is a doctor’s obligation to enhance, empower and enrich the patients’ (and/or the care-takers’) capacity to participate in the decision-making process of their care [28]. Nevertheless, the KNH clinicians as well as other health professions maintained primacy in the care of patients and protected their expert professional role. This power imbalance made patients vulnerable - they did not have any opportunity to question decisions in care such as not being given the prescribed treatment or not being reviewed regularly.

### Institutional pressures led to mal-adaptation

As a teaching hospital there was a felt need to expose the students to a wide spectrum of medical conditions ranging from those that are common to those that are rare. However, it was practically very difficult to adhere to KNH’s vision. Patients with uncomplicated acute illnesses were admitted to KNH due to limited affordable alternative in-patients health facilities and an inadequately functioning referral system. Thus, KNH served primarily as a large acute care facility for low-income families. This reality contrasted with the strategic planning that determined the hospital’s structure, staff recruitment and resource allocation policies that focused on the aspirations of KNH. As a result, there seemed to be a mismatch between the skills and interests of the consultants and the tasks they were faced with. A large number of sub-specialists were not motivated by excellence in management of common illnesses and generally did not consider skills in management of service delivery a priority. Exacerbating this situation the subspecialists, faced with poor resources and congested wards full of children with common illnesses, often felt unable to perform satisfactorily in a subspecialist role. Feeling let down they ultimately rejected the more general role confronting them.

## Implications based on root cause analysis

Adoption of new, best-practices met variable success. We found it useful to adopt a more system-wide perspective and explore the complexity of the barriers and the context in which solutions must be implemented. Tackling root causes of failure takes longer and the solutions are more difficult to advance, but without them, solutions such as training and increased funds often advanced as simple interventions to correct inadequacies are likely to lead to only limited success [29]. We now discuss the implications of our findings in promoting uptake of best practices under three headings: i) improving medical education, ii) improving hospital leadership, and iii) improving institutional collaboration.

### i) Improving medical education

Medical professionalism has for a long time been presumed to be a *calling* supported by attitudinal competency and based on innate characteristics or an altruistic personal philosophy. There is increasing evidence that professionalism cannot be assumed; it is acquired and thus must be taught [30]. In the Kenyan health system where considerable reliance is still placed on professionalism, the starting point for its development should be the undergraduate medical programme, subsequently reinforced by postgraduate programmes and continuous professional development. Cruess *et al*. suggest concepts to be included such as: altruism and the notion of calling, knowledge of the code of ethics, understanding the nature and limitations of individual and collective autonomy and making explicit links between professional status and societal obligations [30]. Thus medical education should pay greater attention to the following:

#### Enhancing adaptation to change

Medical education should enhance capability (the extent to which individuals can adapt to change), generate new knowledge and develop individuals who continue to improve their performance as professionals despite stressors and competing personal and professional priorities [31]. Thus, the teaching methods used in medical schools should enhance creativity and the imaginative dimension of professionals’ capability rather than relying on planned formal events with tightly defined content-orientated learning objectives (an approach replicated in our own intervention during the PAR). Ward-rounds provide chances for this because cases are presented in their real-world context [21]. In particular, ward-rounds can enhance the imaginative dimension of professional capability and help in the development of problem-solving capabilities that contribute to abilities in analytic thinking in complex decision-making processes [17]. However, problem-based learning does not by itself improve knowledge content as assessed by written examinations, thus content learning is required too [21].

#### Inculcating a culture of team-work

Teaching methods used in medical schools should reinforce the culture of team work rather than promote values based on individual decision-making as a collectivistic culture is needed to improve performance in group-oriented activities. Developing team approaches may particularly help people perform if their psychological orientation is congruent with the structure of the social systems in which they work [18].

#### Emphasizing ethics and professionalism

Building professional ethos and identity should be a key role of medical education with implications for the curriculum design and content. This should include teaching leadership skills (see below), communication skills and nurturing ethical sensitivity by adopting a life-long regular practice of reflective learning. The students should also be familiar with the ethical codes and local ethical guidelines that are set by the respective regulatory bodies.

#### Leadership skills

Leadership skills that need to be learned include: leading and motivating teams, networking and appreciating the contributions of others, conflict management, communication and negotiation skills, and planning and organizing meetings. It follows then, that doctors in training and trainee paediatricians should be acquainted with the diagnostic and analytic skills to guide implementation of quality initiatives. These might include developing goals and minimum standards, using clinical audit and feedback as a means to promote reflection, team learning, and using data to understand quality of care and root cause analysis. Emphasis should be on the common aspects of care; processes that affect the majority of patients rather than creating an impression that an effective solution to health care must be complex or reliant on advanced technology. In addition, students should be encouraged to be curious rather than dogmatic, to be inquisitive rather than judgmental, to be empowering rather than patronizing, and to be approachable rather than presenting themselves as someone who knows it all [32].

### ii) Improving hospital leadership

A hospital is a complex system complicated by the fact that it is also a professional organization. Often little thought has been given in low-income settings to leading and managing hospitals and greater attention should be paid to the following:

#### Understanding the characteristics of complex adaptive systems

The hospital’s adaptation, as an organization, to changes as a result of the introduction of quality initiatives was a challenge. It appeared that on one hand, the managers believed they were dealing with learned colleagues who should have regulated themselves and automatically given the best medical care. On the other hand, the consultants, as well as other professionals, expected the hospital managers to supervise and direct them, roles that challenge the professionals’ capability. Given this tension, hospital leadership should enhance a learning culture and adaptation by: i) focussing on relationship building and strategies of sense-making that allow members to construct a shared way of interpreting complex activities and provide the staff with identity and cohesion, ii) recognizing that complex systems have emergent properties that can be stifled by rigid structures, thus there should be a search for improvisational behaviour that enables innovation and creativity at all levels. This should be allied with awareness that interdependencies and interactions within the organization may generate unanticipated, unplanned events that may derail attempts to plan, direct or control [33], and iii) nurturing system thinking – to see the systemic whole and to understand how members’ interactions trigger a network of events [33].

#### Vision alignment

A vision that is merely rhetoric fails to articulate the uniqueness of the organization, provide the basis for the leader to motivate followers or provide a sense of identity. A vision should provide people with a framework for coordination and integration of their activities and provide a foundation for organizational norms and structures [34]. The hospital should confront the gap between its aspirational vision and its reality and generate a more accommodating vision in which it can realign organizational policies and structures with practices. For example, it might benefit from realigning its workforce with one commensurate to the local morbidity and mortality patterns so it is more responsive to societal needs. This will require considerable diplomacy as the allegiance of professionals is most likely to their own profession and societal values rather than to the vision and values of the organization [35].

#### Promote positive professional relationships

Leadership should realize that medicine is teamwork and create a shared orientation for the staff and organization. To enhance team-work, members should understand the scope and limits of the responsibilities of the team members as agreed upon at the operational level. The diversity of the professionals within a hospital should be seen as strength. Interprofessional learning should be strengthened, aimed at building relational networks and more participative decision-making in addressing organizational challenges across and between departments while encouraging emergence of bottom-up innovative solutions that are more adaptive to the local context [33]. This may allow staff to identify with the hospital services and increase individual agency as well as improve their collective capacity to adapt.

#### Inculcate a culture of quality care

The hospital leadership should promote accountability by facilitating professionals to define what practices constitute quality care and subsequently foster development of goals and standards of patients’ care at all levels. The professionals should be facilitated to develop control measures that are embedded in the routine work-flow processes and that promote adherence to standards. Hospitals in similar contexts should consider embracing biomedical ethics with a focus on patients’ interests and the process of shared decision-making rather that a general assumption that professionals serve the best interests of their patients [36]. Health information systems also need to be designed to meet hospital’s needs and provide data that can be used to inform quality improvement decisions embedding these values within a learning organization.

#### Decentralizing power and control

In a complex system, devolution of power enhances adaptability and self-organization [33,35]. In addition, critical analysis of power relationships in centralized administrative systems suggests the irresponsibility and apathy observed among staff in such a system can be addressed by promoting a wider diffusion of power and responsibility through democratization of institutional activities [37]. This can bring professionals and managers into working relationships that promote improved service provision. Further, team leaders should be identified based on their capabilities. This is in contrast to just considering the portfolio of what one has learned; a strategy that creates a situation in which senior staff are retained in *powerful positions* regardless of their capability and efficiency.

### iii) Institutional collaboration

The partnership of a teaching hospital and medical schools creates the social and cultural context in which medicine is practiced and so both share responsibility for tackling the challenges to medical professionalism and their health system consequences. While multiple stakeholders can be a source of strength, they may also complicate the development of solutions linked to the self-interests of parties, competing institutional priorities and societal pressures. This study suggests that collaboration between a hospital and its legitimate partners is one of the fundamental challenges that make the functions of a hospital complex. Solutions must therefore embrace the complexity of the situation, posing an adaptive challenge that requires changes in the individual and collective values of all partners, and in shared mental models of what are good medical and organizational practices.

Collaboration is built on honest dialogue and bilateral access to information between the partners. In addition, to facilitate resource exchange and to clarify changing institutional priorities there should be regular dialogue between the partners. For a collaboration to have optimal *whole*-*system* results, the partners must be willing to invest in the best interests of the *whole*. Tackling complex challenges in an organization is difficult, if not impossible, when part of the organization is immune to stated values, goals and guiding principles. Indeed without exceptional leadership at the top, the immune group can bring even the more well-intended organization to a standstill [38]. Suggested approaches of enhancing collaboration include facilitating staff to take part in negotiation seminars, cheerleading workshops, or team building retreats [38]. But organizations can only do so much, change cannot be mandated or legislated and each individual must be committed to change.

## Conclusion

Mixed methods research and interpretive integration of the results of the research paradigms provided an understanding of the root causes of problems of uptake of CPGs that would not have been obtained if the research methods were applied independently. The findings illustrate that implementation of best-practices is a complex process that is largely unpredictable. This is attributed to the complexity of contextual factors. There is no simple solution to implementation of best-practices; rather solutions require system-wide approaches that take into account the interrelatedness of agencies, functions and other components of the system. Tackling root causes of inadequate knowledge translation will require complex solutions that entail long-term plans, with emphasis on the promotion of professional ethics and values and establishing an organizational framework that enhances the positive aspects of professionalism. Creating such a framework will have major implications for the approach to medical education and the hospital’s vision and leadership. Apparently simple solutions such as dissemination of CPGs, in-service training, and reinforcement activities will likely have limited success without this more fundamental change.

## Abbreviations

CPGs: Clinical practice guidelines; ETAT+: Emergency, triage, assessment, treatment PLUS admission care; KNH: Kenyatta National Hospital; PAR: Participatory action research; UoN: University of Nairobi.

## Competing interests

The authors declare that they have no competing interests.

## Authors’ contributions

GI conceived the idea for this study and its design with advice from ME. ME obtained the funding for this project. The study hospital and ME provided financial support for the guideline dissemination. GI, HK, CM, SM, DM and ME guided/facilitated the quality initiatives in the action research. GI was responsible for qualitative data collection. GI, DG, ME, DZ and JT were responsible for quantitative data collection and analysis. GI, ME, DZ and AG were responsible for interpretive integration of the qualitative and quantitative data sets. GI prepared the initial draft manuscript. All authors reviewed the draft manuscript and provided input to and approval for the final version of the report.

## Authors’ information

CM, HK, DM, ME and GI are paediatricians and legitimate members of KNH and UoN. ME is a Senior Research Fellow with Wellcome Trust, he conceived and was instrumental in development of the CPGs and ETAT+. GI participated in the development of the guidelines and ETAT + training. CM and DM were head of KNH and UoN departments of paediatrics respectively. AG is an anthropologist. DZ and DG are epidemiologists. JT is Biostatistician. SM is a paediatrician and a senior officer in the Ministry of Health.

## Pre-publication history

The pre-publication history for this paper can be accessed here:

http://www.biomedcentral.com/1472-6963/14/119/prepub
